# Microstructure and Pitting Corrosion of Austenite Stainless Steel after Crack Arrest

**DOI:** 10.3390/ma12244025

**Published:** 2019-12-04

**Authors:** Zhuwu Zhang, Guangguo Pan, Yan Jiang, Song Chen, Song Zou, Wei Li, Chengwei Xu, Jingwei Zhang

**Affiliations:** 1 Oil and Gas Storage and Transportation Engineering Department, College of Chemical Engineering, Fuzhou University, Fuzhou 350116, China; panguangguo001@163.com (G.P.); zou.song@cxtc.com (S.Z.); wei.li46@yanfeng.com (W.L.); wc10828646@163.com (C.X.); zhjw@fzu.edu.cn (J.Z.); 2 The First Sector of Fuzhou Department, Fujian Institute of Boiler and Pressure Vessel Inspection Research, Fuzhou 350008, China; jiangyan1201@hotmail.com (Y.J.); s.chen_hit@163.com (S.C.); 3 Quality Inspection Center and Equipment Management Department, Fujian Institute of Boiler and Pressure Vessel Inspection Research, Fuzhou 350008, China

**Keywords:** austenitic stainless steel, crack arrest, nano-indentation, scanning microelectrode technology (SMET), pitting corrosion

## Abstract

With synergy of plastic deformation near crack tip and pulse current treatment, complex phase transformation and recrystallization occur in the metallographic structure, with the austenite transforming to fine grain structure and deformation-induced martensite; but, without the plastic deformation, the phase transformation, and recrystallization it was difficult for the crack arrest process to take place only undergoing the pulse current treatment. The nano-indentation experiment showed that the phase transformation region contained the maximum residual compressive stress consisting of four parts: the plastic stress, the explosion stress, the thermal stress, and the transformation stress, which was beneficial to restrain the crack growth. However, the solidification structure and the deformation-induced martensite structure was vulnerable to pitting corrosion through scanning microelectrode technology (SMET) and pitting corrosion experiment, but the pitting corrosion resistance could be improved through the solution heat treatment.

## 1. Introduction

Crack is one of the most common failure modes of metal components, frequently leading to serious accidents and damages. If the crack propagation is prevented by effective means, reliability and service life of the components would be significantly improved. Theoretical studies and experiments have shown that the crack propagation could be suppressed effectively using electromagnetic heat effects [1,2,3]. When a high-voltage pulse current was loaded on a metal component with a crack, the current would flow around the crack tip, causing large amount of charge accumulation and instantly generating huge heat. As a result, the temperature at the crack tip would rise to melting point of the metal and form a melting hole, which is beneficial to crack arrest.

In recent years, researches about the crack arrest mainly focused on the temperature field and stress field distribution. For instance, a theoretical model for electromagnetic heating crack arrest process of space cracks in metal die has been established, indicated that the process could reduce stress concentration, and formed a compressive stress region at the crack tip, and inhibited the crack propagation [4,5]. Moreover, the thermoelectric coupling field of metal plates with circular holes and through cracks have been studied, implied that temperature at crack tip depended on the electrical energy input, geometry and mechanical load [6,7].

In addition, some scholars have paid attention to metallographic structure after the crack arrest, and pointed out that extremely fine recrystallized structure near crack tip was favorable to resist crack formation and expansion after the pulse current treatment [8], and also found that refined grains and sub-grains were observed in fracture crack tip of the as-cast hot-worked die steel after current treatment, and the dislocation density gradually increased [9].

However, mechanism of the metallographic transformation is also unclear in the heat affected zone near crack tip in the crack arrest process. Moreover, corrosion resistance of materials, especially austenitic stainless steel, near crack tip has been rarely reported after the pulse current treatment. According to the theory of fracture mechanics, a plastic zone would be formed near crack tip in the process of crack growth, which had a significant influence on the metallographic transformation and the corrosion property of austenitic stainless steel.

Although austenitic stainless steel has good corrosion resistance, it is susceptible to pitting corrosion in a solution containing Cl^−^. Because of small range of the heat affected zone near crack tip, traditional electrochemical corrosion research methods, such as anodic polarization curve and electrochemical impedance spectrum, are difficult to conduct local corrosion research. However, scanning microelectrode technology (SMET) has been used to detect in situ the electric field distribution at the metal/solution interface, to reveal the position and potential of electrochemical activity points in the two-dimensional direction of metal surface, and to track the changes of activity points [10]. SMET has also been successfully applied to study various forms of local corrosion [11,12,13,14], such as mechanism and development process of pitting corrosion and crevice corrosion, and to evaluate local corrosion resistance of materials. Therefore, much important information could be obtained using SMET, but difficultly using general electrochemical methods.

In this paper, optical microscopy (OM), scanning electron microscopy (SEM), nano-indentation, SMET were used to study the microstructure, residual stress, and pitting corrosion near crack tip of 304 stainless steel specimens with plastic zone or non-plastic zone after the pulse current treatment. Moreover, influence of solution heat treatment for material properties near crack tip was also considered. The mechanisms of metallographic transformation and residual stress change near crack tip of 304 stainless steel were analyzed in detail. The reasons for change of the pitting corrosion resistance in different micro-zones near crack tip were discussed.

## 2. Materials and Methods

The chemical composition of the 304 stainless steel plate with a thickness of 1 mm was shown in Table 1.

**Figure 1:**
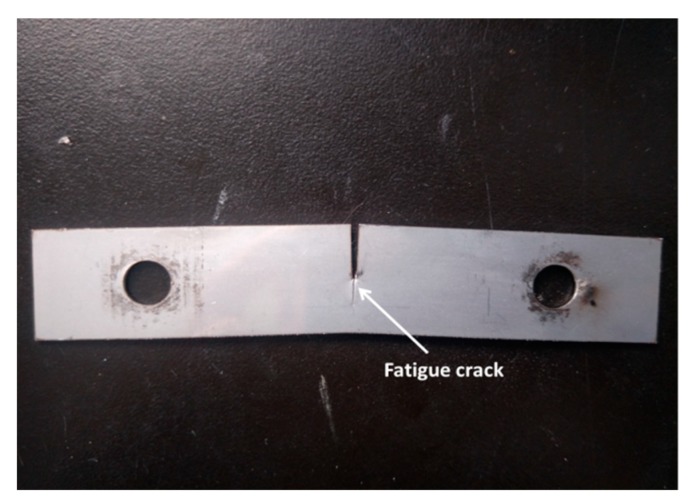
Specimen with a crack.

**Figure 2:**
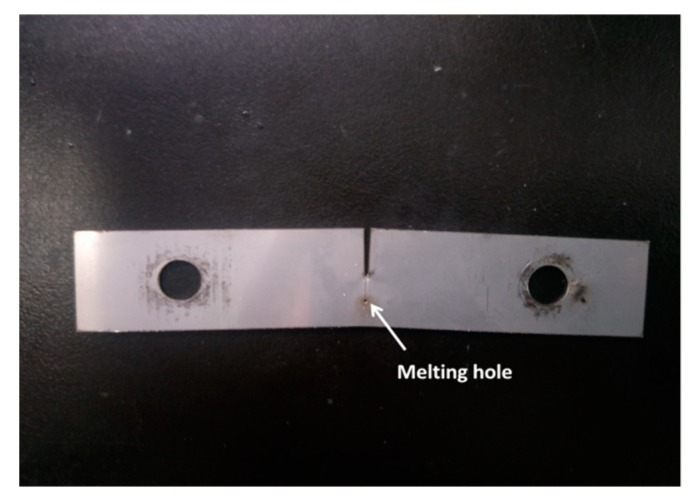
Specimen after crack arrest.

The 304 stainless steel plate was cut into specimens with a size of 110 mm × 20 mm × 1 mm, and with a gap of 10 mm in length using wire-electrode cutting in the middle of the specimens grouped as A, B, C, and D. Then the fatigue cracks of a certain length were made using hydraulic servo fatigue tester. A specimen with a crack was showed in Figure 1. Specimen A only prefabricated fatigue crack without any other treatment; specimen B with a prefabricating fatigue crack was treated using plus current to form a melting hole at the crack tip. The pulse current was applied at both ends of the specimen in a direction perpendicular to the crack, so that the crack tip exploded to form a melting hole reaching crack arrest target. A specimen after crack arrest was showed in Figure 2. The crack arrest was carried out using pulse current experiment platform. Specimen C underwent solution heat treatment after prefabricating fatigue crack, and then was treated using plus current to form a melting hole at the crack tip. The heat treatment was carried out in a box atmosphere furnace with the temperature 1050 °C and holding 30 min, and then the specimen was removed from the heat treatment furnace and cooled with water immediately. Specimen D with a prefabricating fatigue crack was treated using plus current to form a melting hole at the crack tip, and then underwent solution heat treatment. The specimens were cut with a square of 10 mm × 10 mm with the crack tip or the melting hole as the center. The square specimens were polished successively with 180, 240, 600, 800, 1000, 1200 grade SiC paper and then to a mirror finish using 2.5-μm and 1-μm diamond powder. Finally, the specimens were washed with ethanol water for 10 min, and then dried and placed in a drying vessel.

**Figure 3:**
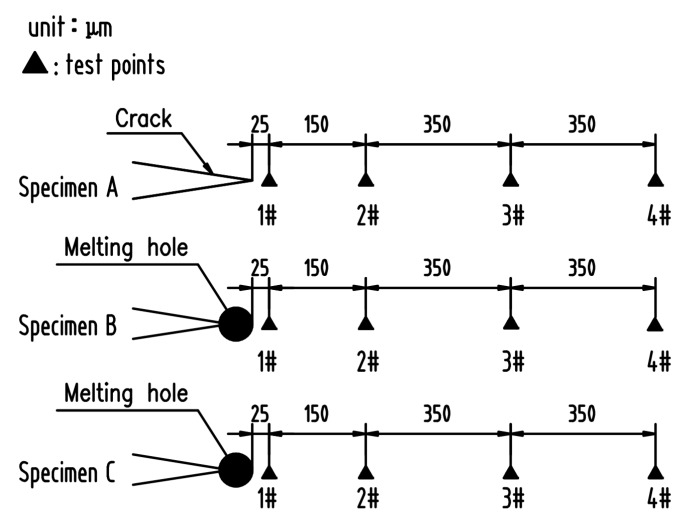
Distribution of nano-indentation test points.

Metallographic observations were performed using OM and FEI QUANTA 250 SEM (FEI, Hillsboro, OR, USA). The specimens were electrolytically etched with 50% nitric acid solution. The Anton Paar NHT2 nanometer indentation tester (Anton Paar, Corcelles, Switzerland) was used to measure the residual stress near the crack tip or melting hole. The distribution of measurement points is shown in Figure 3.

**Figure 4:**
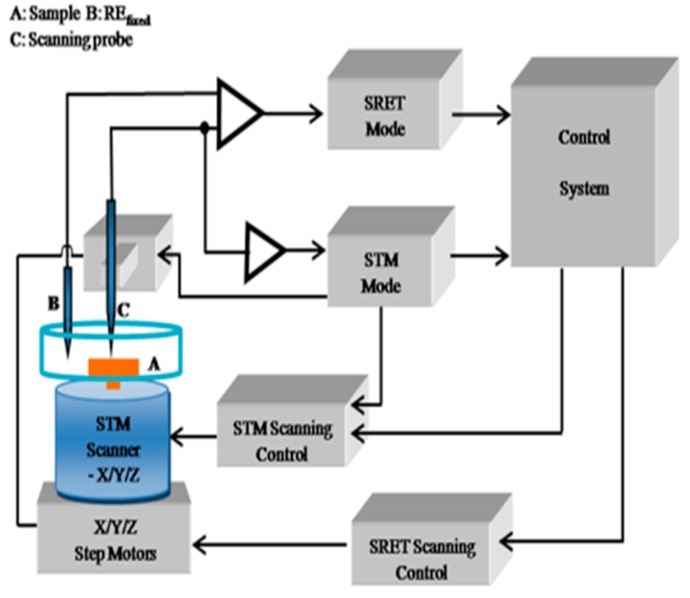
The schematic diagram of the scanning system.

The pitting corrosion test near melting hole was carried out on the XMU-BY scanning electrochemical workstation produced by Xiamen Legang Material Technology Co., Ltd. (Xiamen, China) (Figure 4). Prior to the experiment, 10 mm × 10 mm square specimens (including the melting hole) were spot-welded with a stainless steel nut, and then were covered with epoxy resin except for testing surface. In scanning tunneling microscope (STM) mode, the probe was automatically placed within 1 nm of the specimen surface, and then the probe was retracted by 500 steps (50 nm per step) using a three-dimensional platform. The scanning parameters were set on the micro-area potential platform, and the scanning area was 3 mm × 3 mm, and the scanning frequency was 0.4 Hz, and the signal amplification factor was 100 times. A 10% FeCl_3_ solution was added to the corrosion bath until the specimen was completely immersed, and then the surface potential near the melting hole of the 304 stainless steel was scanned.

## 3. Results and Discussion

### 3.1. Analysis of Microstructure Morphology near Crack Tip or Melting Hole

**Figure 5:**
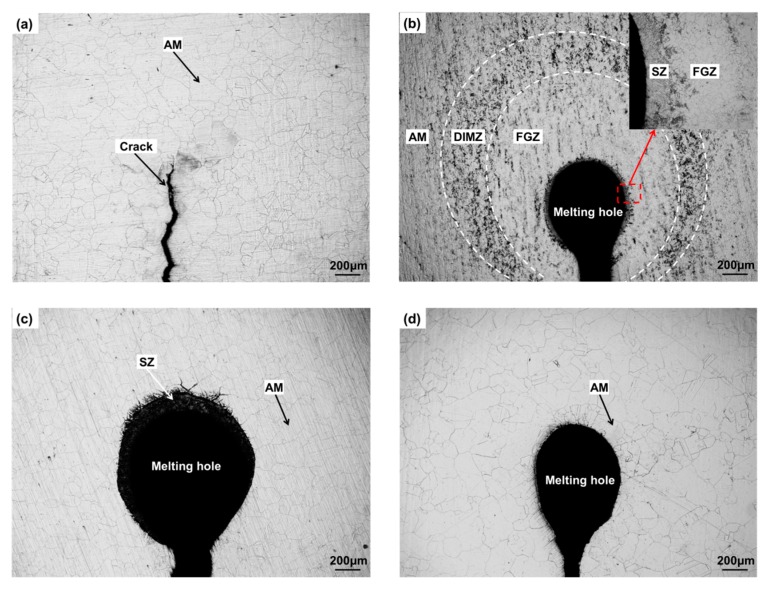
Photos of metallographic structure near crack tip or melting hole. (**a**) Specimen A, (**b**) Specimen B, (**c**) Specimen C, (**d**) Specimen D.

The metallographic structure near the crack tip or melting hole of specimen A, B, C, and D is shown in Figure 5. The metallographic structure near the crack tip of specimen A was austenite matrix (AM) (Figure 5a). As shown in Figure 5b, specimen B formed a melting hole after pulse current treatment, because temperature at the crack tip exceeded the melting point of 304 stainless steel attributed to the current concentration around the crack tip. In addition, solidification zone (SZ), fine grain zone (FGZ), deformation-induced martensite zone (DIMZ), and AM was observed successively with the increase of distance from the melting hole edge. The metallographic structure of specimen C near melting hole are only SZ and AM, because the specimen C has undergone solution heat treatment before the crack arrest which eliminated the residual stress of the plastic zone near melting hole (Figure 5c). The metallographic structure of the specimen D is AM near melting hole, because the different microstructure zones around melting hole has been eliminated in the process of solution heat treatment after the crack arrest.

The solidification zone could be observed around the melting hole if the specimens, such as B and C, underwent pulse current treatment resulting in temperature of the crack tip reaching melting point, but could be eliminated through the solution heat treatment, i.e., specimen D. The plastic zone formed in the process of prefabricated fatigue crack, i.e., specimen A, but no FGZ and DIMZ were observed near crack tip, indicated that the plastic deformation near crack tip was insufficient to cause the martensite transformation. In addition, FGZ and DIMZ were also not observed in specimen C, implied that the thermal stress generated by temperature field after the pulse current treatment was also not enough to cause the recrystallization and the martensite transformation near melting hole. However, FGZ and DIMZ could be observed in specimen B, illustrated that the recrystallization and the martensite transformation were the result of combined action of the plastic deformation near the crack tip and the pulse current treatment.

**Figure 6:**
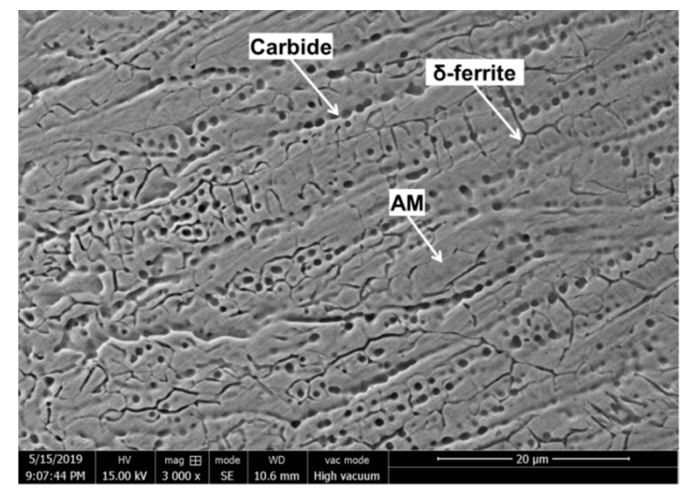
SEM photo of the solidified microstructure.

As shown in Figure 6, the microstructure of the solidification zone was a large number of worm-like δ ferrite and round carbide particles distributing on the austenite matrix. The solidification structure of austenite depended on its solidification mode, which was related to Cr_eq_/Ni_eq_ in steel. The experimental 304 stainless steel had a Cr_eq_ = 18.1 wt.%, Ni_eq_ = 10.5 wt.%, and Cr_eq_/Ni_eq_ = 1.72, according to Equations (1) and (2) proposed by Hammar and Svensson [15]. Therefore, the equilibrium solidification mode should be the FA mode (the solidification sequence is L (liquid) → L + δ → L + δ +γ (γ: austenite) → δ + γ), which meant that the δ ferrite separated out first [16]. However, the temperature near crack tip dropped rapidly resulting in the transformation fr om δ to γ being restrained, attributed to the fast process of pulse current discharge, the small range of heat source near the crack tip and the good heat conduction of the 304 steel. Moreover, during the melting process, a large amount of carbides were dissolved, but in the process of rapidly cooling, the carbides were quickly and extensively nucleated and grew. The large temperature gradient and the short cooling process led to a huge degree of supercooling, which impelled a large nucleation rate of the carbide [8]. However, owing to extremely low growth rate, the final shape of the carbide presented tiny dots on nanometer scale. 

Cr_eq_ = Cr + 1.37Mo + 1.5Si + 2Nb + 3Ti (1)

Ni_eq_ = Ni + 22C + 14.2N + 0.31Mn + Cu (2)

The fine grain structure, an austenite recrystallized structure, existed only near the melting hole of specimen B (Figure 5b), the driving force of recrystallization from the plastic deformation near the crack tip and the pulse current treatment. In addition, a large degree of supercooling and thermal stress near the hole were generated because of the fast cooling rate after the pulse current treatment, which restrained the growth of austenite grain resulting in the size of the grains being relatively small.

**Figure 7:**
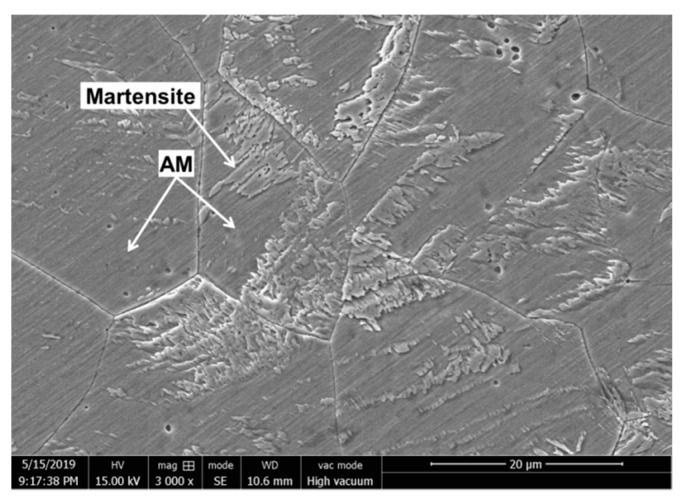
SEM photo of deformation-induced martensite.

The deformation-induced martensite was also found only around the melting hole of specimen B. As shown in Figure 7, microstructure of the deformation-induced martensite was lath martensite distributing on the austenite matrix. The lath martensite was embossed on surface of the austenite matrix. The structure of 304 stainless steel was metastable austenite, thus the face-centered cubic austenite in the plastic zone would transform to the body-centered cubic martensite, when the highest temperature of local region near melting hole was below M_d_ in the process of pulse current treatment (the highest temperature of deformation-induced martensite) [17]. However, if the highest temperature was above the M_d_, it was difficult to induce the martensitic transformation in the plastic zone only through the pulse current treatment, i.e., specimen C.

### 3.2. Residual Stress near Crack Tip or Melting Hole Using Nano-indentation

**Figure 8:**
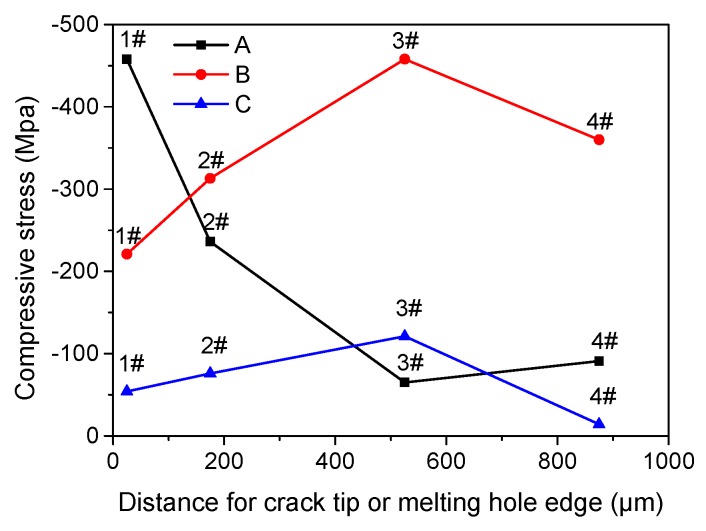
Residual stress distribution of specimen A, B, and C.

Since the compressive stress zones near crack tip or melting hole were extremely small, the conventional methods for residual stress measuring could not achieve sufficient accuracy. The specimen A, B, and C were tested according to Suresh’s residual stress measurement method using the nanometer indentation tester [18]. The compressive stress values near crack tip or melting hole edge detected using the nano-indentation are shown in Table 2. It can be seen that the maximum compressive stress near crack tip of the specimen A was located at 1# point (Figure 8) that meant large plastic deformation there, and that the stress was rapidly attenuated with the increase of distance from the crack tip. The compressive stress values of specimen B rise with the increase of distance from melting hole edge, and the maximum compressive value was located at 3# point in the martensite zone attributed to the martensite phase transformation stress. The compressive stress distribution of specimen C was similar to specimen B, but the compressive stress values were lower than specimen B as a whole.

The residual stress near melting hole came from two ways. One was plastic stress in the plastic zone near crack tip generating in the process of precrack, such as the specimen A. The other one was generated through the pulse current treatment composing of three parts: explosion stress in the process of crack tip melting, thermal stress from the huge temperature gradient in the heat-affected zone, and martensite transformation stress. The residual stress of specimen B was from the both ways consisting of four parts: the plastic stress, the explosion stress, the thermal stress and the transformation stress, but the residual stress of specimen C only consisting of two parts: the explosion stress and the thermal stress leading to lower compressive stress values as a whole.

### 3.3. Pitting Corrosion near Melting Hole

**Figure 9:**
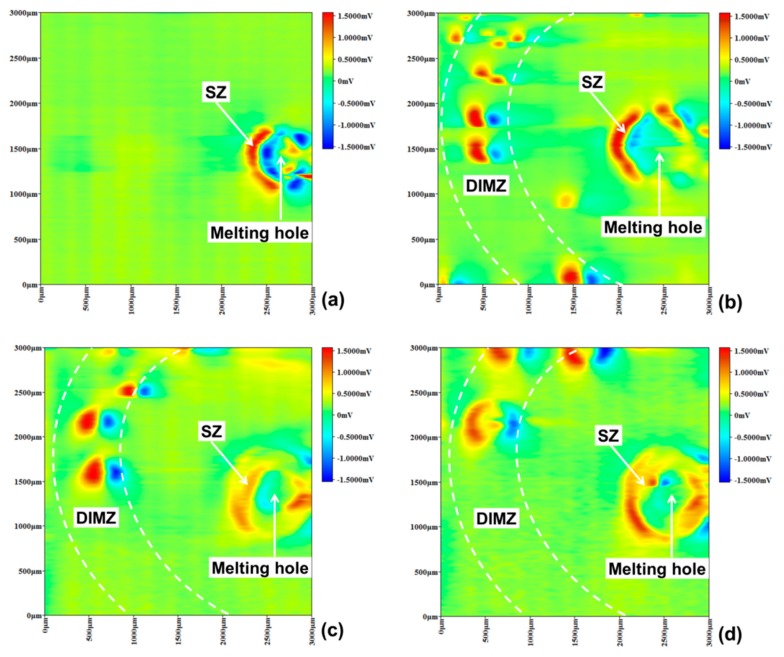
Specimen B micro-area potential distribution using scanning microelectrode technology (SMET). (**a**) 15 min, (**b**) 30 min, (**c**) 45 min, (**d**) 60 min.

As shown in Figure 9, micro-area potential of specimen B changed over time near melting hole immersed in a 10% FeCl_3_ solution using SMET, which indicated different pitting corrosion trend. The points of pitting corrosion were tiny as active anodes, so that the electric field lines were densely concentrated in the points exhibiting high potential peaks in red state. First, after the specimen B was immersed in the solution for 15 min, a high potential region in red could be observed in the solidification zone, indicated the region’s surface passivation state was destroyed and lots of pitting corrosion occurred. As shown in Figure 6, a large amount of δ ferrite and carbide were formed during the solidification of austenitic stainless steel, which easily led pitting corrosion to take place [19,20,21].

Then, after specimen B was immersed for 30 min, the high potential peak area of the solidification zone became larger; what was important that large numbers of high potential peaks appeared in the martensite region meaning that severe pitting corrosion happened in this region. Because, the deformation-induced martensite region had high density dislocations, which would result in energy of the martensite phase higher than austenitic phase [22,23,24]. In addition, as shown in Figure 7, the lath martensite could be observed embossing on the surface of the austenite matrix, which contained many defects such as dislocations, vacancies, interstitial atoms, and stacking faults [25]. The defects and embossing shape would bring about weaker part in passivation film, and more possibly resulted in pitting corrosion caused by Cl^−^, martensite as anode and austenite as cathode in the corrosion process.

When specimen B was immersed for 45 min, the corrosion potential peak of the solidification zone began to decrease, illustrating that this region entered uniform corrosion state. However, the pitting corrosion of the martensite region was still intensive. Finally, with the immersion time extending to 60 min, the potential distribution in the solidification zone did not change much, but the potential peaks of the martensite region declined meaning that the pitting corrosion developed to small area surface corrosion.

**Figure 10:**
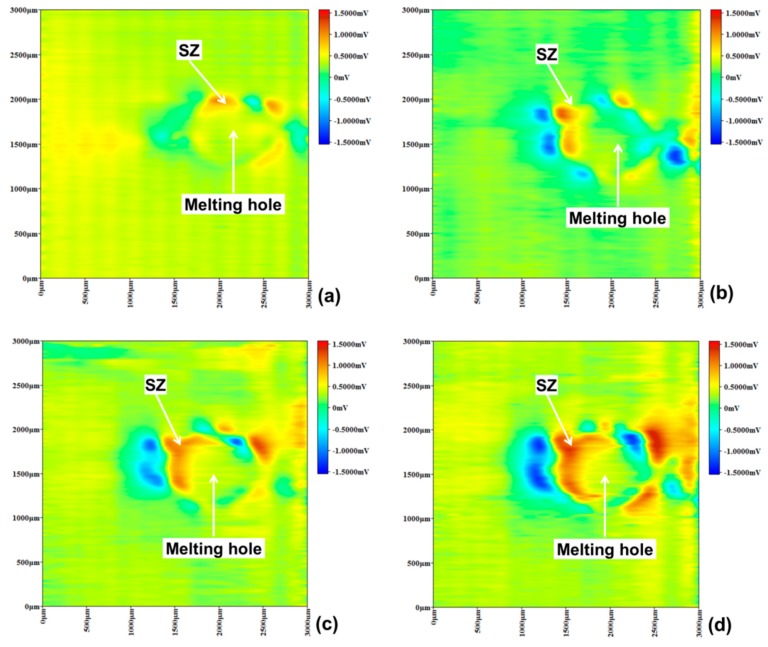
Specimen C micro-area potential distribution using SMET. (**a**) 15 min, (**b**) 30 min, (**c**) 45 min, (**d**) 60 min.

As shown in Figure 10, micro-area potential of specimen C changed over time near the melting hole immersed in a 10% FeCl_3_ solution, indicated that pitting corrosion also occurred in the solidification zone according to the red potential region after 15 min. Then, area of the pitting corrosion in the solidification zone has grown with time, which was similar to specimen B. However, more pitting corrosion has not been observed in other region, implying that the passive film was not destroyed and the martensite transformation did not occur there.

**Figure 11:**
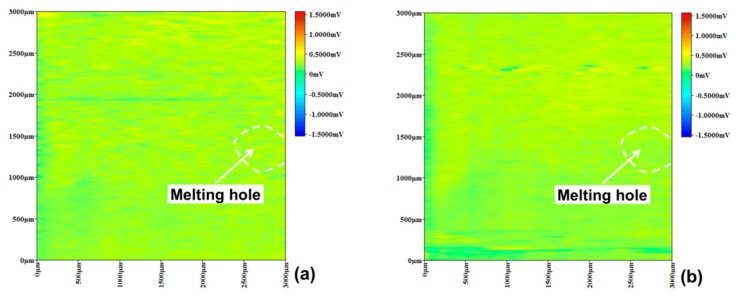

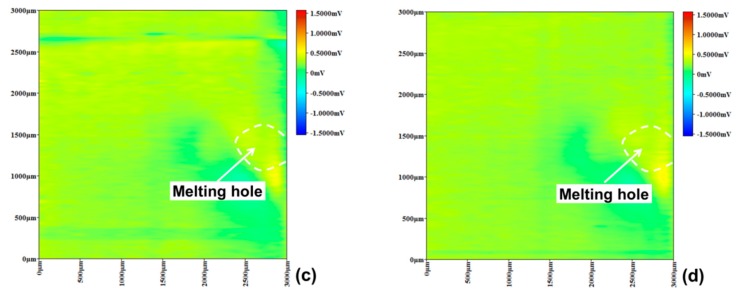
Specimen D micro-area potential distribution using SMET. (**a**) 15 min, (**b**) 30 min, (**c**) 45 min, (**d**) 60 min.

According to uniform potential distribution state, specimen D did not exhibit a high potential peak of pitting corrosion after immersed in a 10% FeCl_3_ solution for 15 min (Figure 11). Then, some small pitting corrosion points have been observed after 30 min, but disappeared quickly with time. Moreover, additional pitting corrosion point has not been observed after being immersed for 60 min, illustrating that the passive film of specimen D maintained integrity and could be self-healed if being destroyed. The results implied that the solution heat treatment could effectively restrain pitting corrosion near the melting hole after the crack arrest.

### 3.4. Pitting Corrosion Morphology in 10% FeCl_3_ Solution

**Figure 12:**
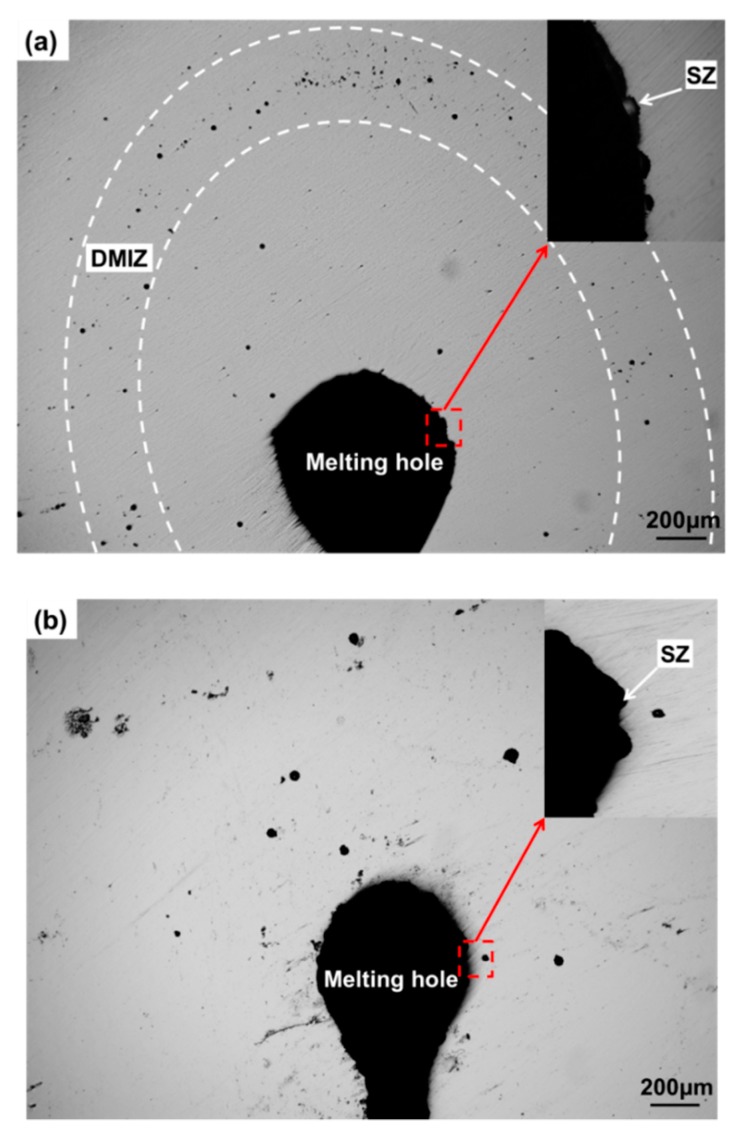

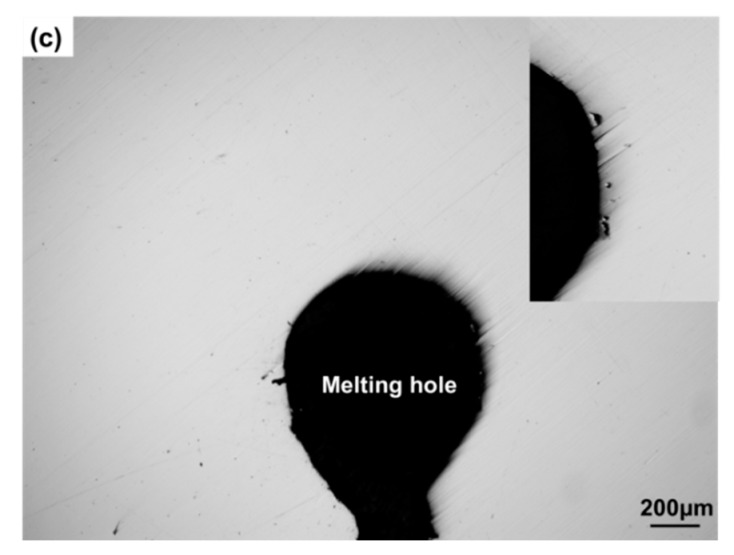
Surface images of specimens after soaking 1 h in 10% FeCl_3_ solution. (**a**) Specimen B, (**b**) Specimen C, (**c**) Specimen D.

A large number of pitting corrosion points could be observed in the martensite region along with the melting hole edge of the specimen B after soaking for 1 h in 10% FeCl_3_ solution (Figure 12a). The specimen C showed different phenomenon that pitting corrosion points were mainly in the solidification zone and randomly distributed around the melting hole attributed to some inclusions or carbides in the stainless steel (Figure 12b). However, no visible corrosion pits could be found in the specimen D, except few corrosion pits at the melting hole edge, because of the solution heat treatment to eliminate asymmetrical metallographic structure of the stainless steel after the crack arrest (Figure 12c). Compared with the potential distribution using SMET as shown in Figure 8, Figure 9 and Figure 10, the pitting corrosion point distribution of the specimens had consistent regularities. 

## 4. Conclusions

If the crack tip of 304 stainless steel existed in plastic zone, the metallographic structure would carry out complex phase transformation and recrystallization after the plus current treatment, consisting of solidification zone, fine grain zone, deformation-induced martensite zone, and austenite matrix zone from the melting hole edge to base metal, such as specimen B; but, if it did not exist, the phase transformation and recrystallization was difficult to happen, the metallographic structure only consisting of solidification zone and austenite matrix zone, such as specimen C. Compared with other specimens, specimen B contained the maximum residual compressive stress consisting of four parts: the plastic stress, the explosion stress, the thermal stress, and the transformation stress, which was beneficial to restrain crack growth.

However, the solidification structure and the deformation-induced martensite structure, attributed to the phase transformation, easily led to pitting corrosion through SMET and the pitting corrosion experiment, which reduced pitting corrosion resistance of the heat affected zone near the melting hole, but the pitting corrosion resistance could be improved through the solution heat treatment.

## Figures and Tables

**Table 1:** Chemical composition of 304 stainless steel (wt.%).

C	Si	Mn	Cr	Ni	P	S	N	Fe
0.03	0.4	2.0	17.5	7.8	0.045	0.03	0.1	Bal

**Table 2:** Residual stress values (MPa).

Specimen	1#	2#	3#	4#
A	−458	−236	−65	−91
B	−221	−313	−458	−360
C	−54	−76	−121	−14
